# Gray/White Matter Contrast in Parkinson’s Disease

**DOI:** 10.3389/fnagi.2018.00089

**Published:** 2018-03-27

**Authors:** Carme Uribe, Barbara Segura, Hugo C. Baggio, Alexandra Abos, Anna I. Garcia-Diaz, Anna Campabadal, Maria J. Marti, Francesc Valldeoriola, Yaroslau Compta, Nuria Bargallo, Carme Junque

**Affiliations:** ^1^Medical Psychology Unit, Department of Medicine, Institute of Neuroscience, University of Barcelona, Barcelona, Spain; ^2^Institute of Biomedical Research August Pi i Sunyer (IDIBAPS), Hospital Clinic, Barcelona, Spain; ^3^Centro de Investigación Biomédica en Red Sobre Enfermedades Neurodegenerativas (CIBERNED), Hospital Clínic de Barcelona, Barcelona, Spain; ^4^Parkinson’s Disease and Movement Disorders Unit, Neurology Service, Hospital Clínic de Barcelona, Institute of Neuroscience, University of Barcelona, Barcelona, Spain; ^5^Centre de Diagnòstic per la Imatge, Hospital Clínic, Barcelona, Spain

**Keywords:** Parkinson’s disease, gray/white matter contrast, cortical thickness, aging, magnetic resonance imaging

## Abstract

Gray/white matter contrast (GWC) decreases with aging and has been found to be a useful MRI biomarker in Alzheimer’s disease (AD), but its utility in Parkinson’s disease (PD) patients has not been investigated. The aims of the study were to test whether GWC is sensitive to aging changes in PD patients, if PD patients differ from healthy controls (HCs) in GWC, and whether the use of GWC data would improve the sensitivity of cortical thickness analyses to differentiate PD patients from controls. Using T1-weighted structural images, we obtained individual cortical thickness and GWC values from a sample of 90 PD patients and 27 controls. Images were processed with the automated FreeSurfer stream. GWC was computed by dividing the white matter (WM) by the gray matter (GM) values and projecting the ratios onto a common surface. The sample characteristics were: 52 patients and 14 controls were males; mean age of 64.4 ± 10.6 years in PD and 64.7 ± 8.6 years in controls; 8.0 ± 5.6 years of disease evolution; 15.6 ± 9.8 UPDRS; and a range of 1.5–3 in Hoehn and Yahr (H&Y) stage. In both PD and controls we observed significant correlations between GWC and age involving almost the entire cortex. When applying a stringent cluster-forming threshold of *p* < 0.0001, the correlation between GWC and age also involved the entire cortex in the PD group; in the control group, the correlation was found in the parahippocampal gyrus and widespread frontal and parietal areas. The GWC of PD patients did not differ from controls’, whereas cortical thickness analyses showed thinning in temporal and parietal cortices in the PD group. Cortical thinning remained unchanged after adjusting for GWC. GWC is a very sensitive measure for detecting aging effects, but did not provide additional information over other parameters of atrophy in PD.

## Introduction

Gray/white matter contrast (GWC) extracted from T1-weighted MRI images is a measure of blurring between the boundaries of these brain compartments. Even though the neurobiological bases of this measure are not well understood, several studies suggested that the GWC could be an indicator of local variations in tissue integrity and myelin degradation (Koenig, 1991), increasing water content in the white matter (WM; Bansal et al., 2013), or iron deposition (Ogg and Steen, 1998). GWC changes have been associated with aging (Davatzikos and Resnick, 2002), showing a pattern of contrast decay mainly in frontal (Westlye et al., 2009) and temporo-parietal regions; results have been similar or even statistically stronger than those obtained using cortical thickness measures (Salat et al., 2009; Westlye et al., 2009). The interpretation of age-related GWC reductions is still a matter of discussion. An early study that measured T1 and T2 signal intensities in gray matter (GM) and white matter (WM) of elderly subjects suggested that these measures could be reflecting an increase in water content in the WM and neuronal loss in GM (Magnaldi et al., 1993). An alternative interpretation is that GWC reflects changes in myelination degree. In a recent elegant study with a large sample of normal subjects, using cortical reconstruction methods to obtain GWC that greatly improve the delineation of the GM/WM boundary (Dale et al., 1999), Vidal-Piñeiro et al. (2016) observed that GWC seems especially related to age-related myelin variations underlying the GM/WM boundary of lightly myelinated areas. The authors proposed that GWC might be a useful technique to track myelin breakdown in critical brain areas in clinical populations.

Consequently, GWC could be an indirect marker of changes in histological properties of the brain that might have a significant impact on neurodegenerative processes. In Alzheimer’s disease (AD) patients, cortical thickness analyses after adjusting for GWC have shown regionally enlarged and strengthened results in inferior and superior parietal gyri; precuneus; and medial and lateral frontal as well as temporal regions. It has been suggested that this procedure probably corrects for the overestimation of thickness in subjects with regionally reduced tissue contrast (Westlye et al., 2009). GWC has also been proven useful to detect patients with mild cognitive impairment who will later convert to dementia after adjusting this measure for cortical thickness, hippocampal volume, APOE4 status and scanner type (Jefferson et al., 2015).

In Parkinson’s disease (PD), magnetic resonance imaging (MRI) has revealed cortical atrophy mainly through voxel-based morphometry and cortical thickness measures (Hall et al., 2016; Agosta et al., 2017) and WM changes using diffusion tensor imaging (Hall et al., 2016). However, to the best of our knowledge, there are no previous studies on GWC. Therefore, the aims of our study were to test: (1) whether GWC is sensitive to aging changes in PD patients; (2) whether PD patients differ from healthy controls (HCs) in GWC; and (3) whether GWC data would improve the sensitivity of cortical thickness analyses to differentiate between PD patients and controls.

## Materials and Methods

### Participants

The study included 121 consecutive PD patients recruited from an outpatient movement disorders clinic (PD and Movement Disorders Unit, Department of Neurology, Hospital Clinic, Barcelona, Catalonia, Spain) and 49 HC who volunteered to take part in studies addressing age-related processes at the *Institut de l’Envelliment* (Aging Institute). The inclusion criteria considered by the neurologists involved (MJM, FV and YC) were: (1) fulfilling the UK PD Society Brain Bank diagnostic criteria for PD (Hughes et al., 1992) and (2) no surgical treatment with deep brain stimulation. The exclusion criteria were: (1) presence of dementia according to the Movement Disorders Society criteria for PD (Emre et al., 2007); (2) Hoehn and Yahr (H&Y) scale (Hoehn and Yahr, 1967) score greater than 3; (3) juvenile-onset PD; (4) presence of psychiatric or neurological comorbidity; (5) low global intelligence quotient estimated by the Vocabulary subtest of the Wechsler Adult Intelligence Scale, 3rd edition (scaled score 7 points); (6) Mini-Mental state examination (Folstein et al., 1975) score below 25; (7) presence of claustrophobia; (8) pathological MRI findings other than mild WM hyperintensities in long-TR sequences; and (9) MRI artifacts. Ninety PD patients and 27 healthy volunteers were finally selected. Twelve patients and eight controls were excluded because they fulfilled criteria for dementia or other neurological disease, six PD patients for psychiatric comorbidity, one PD patient who scored higher than 3 on the H&Y scale, one PD patient with young-onset PD, three PD patients and one control who had low global intelligence quotient scores, two PD patients for claustrophobia, three healthy subjects who did not complete the protocol, and two controls and two PD patients because of MRI artifacts. Five controls were excluded because of preprocessing errors detected by FreeSurfer. We also excluded four patients and three controls aged younger than 50 years.

This study received approval by the ethics committee of the University of Barcelona (IRB00003099). Written informed consent was obtained from all study subjects after full explanation of the procedures involved.

### Clinical and Neuropsychological Assessment

Motor symptoms were assessed by means of the UPDRS-III motor section (Elton, 1987). H&Y staging and disease duration was also recorded. All PD patients were taking antiparkinsonian drugs, consisting of different combinations of L-DOPA, catechol-O-methyltransferase (COMT) inhibitors, monoamine oxidase inhibitors, dopamine agonists, and amantadine. In order to standardize doses, the L-DOPA equivalent daily dose (LEDD; Tomlinson et al., 2010) was calculated.

We used a neuropsychological battery following the Movement Disorders Society task force recommendations (Litvan et al., 2012), bar language, for which a single measure was used, and executive functions, for which phonemic and semantic verbal fluency were used as two distinct proxies. Detailed information can be found in a previous study by our group (Uribe et al., 2016).

### Image Acquisition and Preprocess

Magnetic resonance images were acquired with a 3T scanner (MAGNETOM Trio, Siemens, Germany). The scanning protocol included high-resolution three-dimensional T1-weighted images acquired in the sagittal plane (TR = 2300 ms, TE = 2.98 ms, TI = 900 ms, 240 slices, FOV = 256 mm; matrix size = 256 × 256; 1 mm isotropic voxel) and an axial FLAIR sequence (TR = 9000 ms, TE = 96 ms).

Cortical thickness was estimated using the automated FreeSurfer stream (version 5.1; available at: https://surfer.nmr.mgh.harvard.edu/). The procedures carried out by FreeSurfer include removal of nonbrain data, intensity normalization (Fischl et al., 2001), tessellation of the GM/WM boundary, automated topology correction (Dale et al., 1999; Ségonne et al., 2007) and accurate surface deformation to identify tissue borders (Dale and Sereno, 1993; Fischl and Dale, 2000; Fischl et al., 2002). Cortical thickness is then calculated as the distance between the WM and GM surfaces at each vertex of the reconstructed cortical mantle (Fischl et al., 2002). In our study, results for each subject were visually inspected to ensure accuracy of registration, skull stripping, segmentation and cortical surface reconstruction. Maps were smoothed using a circularly symmetric Gaussian kernel across the surface with a full width at half maximum (FWHM) of 30 mm.

Intensity maps for WM and GM were created using the values of the T1-weighted signal of the WM at 1 mm below the white surface, and the values of the GM were taken at half of the thickness of the cortex. Finally, WM/GM intensity contrast maps were calculated using the percentage contrast between WM and GM intensities. Before performing group statistical analyses, the resulting WM, GM and WM/GM contrast maps were mapped to a common surface and smoothed with a 30 mm Gaussian kernel of FWHM^1^.

### Statistical Analyses

Demographic, neuropsychological, and clinical statistical analyses were conducted using IBM SPSS Statistics 24.0 (IBM Corp., Armonk, New York, NY, USA). Student’s *t*- or Mann-Whitney’s U tests were used for testing group differences in demographic and clinical variables.

Multiple regression analyses were performed to test the relationship between GWC and age, years of disease evolution, age of disease onset, UPDRS-III and LEDD. Intergroup cortical thickness and GWC comparisons were performed using a vertex-by-vertex general linear model with FreeSurfer. The model included cortical thickness or GWC as a dependent factor and group as an independent factor. A second model included cortical thickness as the dependent factor and GWC as a nuisance covariate. All results were corrected for multiple comparisons using precached cluster-wise Monte Carlo simulation with 10,000 iterations. Reported cortical regions reached a two-tailed corrected significance level of *p* < 0.05. Different cluster-forming thresholds were applied at *p* < 0.05, *p* < 0.01 and *p* < 0.0001 with the aim to identify specific regional correlations between age and GWC.

## Results

Table 1 shows the sociodemographic and clinical data of the groups and the corresponding group comparison. There were no significant differences between groups in age, sex, or education. PD patients scored significantly lower than HC in MMSE. Forty-four percent of the patients had mild cognitive impairment, being the attention and working memory the most frequently impaired domains (59%), followed by the memory domain (40%). Executive functions were impaired in 33% of the patients, visuospatial functions in 30%, and the language domain in 8% of the PD sample.

**Table 1:** Demographic and clinical data.

	PD (*n* = 90)	HC (*n* = 27)	Stats (*p* value)
Sex, male/female	52/38	14/13	0.297 (0.586)^a^
Age, years, mean ± SD	64.4 ± 10.6	64.9 ± 8.4	0.236 (0.814)^b^
Education, years, median ± IQ range	10.0 ± 8.0	9.0 ± 9.0	1098.0 (0.447)^c^
MMSE, median ± IQ range	29.0 ± 1.0	30.0 ± 1.0	841.5 (0.009)^c^
Disease duration, years, median ± IQ range	6.0 ± 9.0	NA	NA
Age of onset, years, mean ± SD	56.3 ± 11.6	NA	NA
UPDRS part III, median ± IQ range	13.0 ± 13.0	NA	NA
Hoehn and Yahr stage, *n* 1/1.5/2/2.5/3	22/5/44/9/10	NA	NA
LEDD, mg, median ± IQ range	700.0 ± 833.0	NA	NA
Mild cognitive impairment, *n* (%)	40 (44.4)	NA	NA
Visuospatial functions, *n* (%)	27 (30.0)	NA	NA
Executive functions, *n* (%)	30 (33.3)	NA	NA
Memory, *n* (%)	36 (40.0)	NA	NA
Attention and working memory, *n* (%)	53 (58.9)	NA	NA
Language, *n* (%)	7 (7.8)	NA	NA

*Abbreviations: HC, healthy controls; IQ range, interquartile range; LEDD, L-DOPA equivalent daily dose; MMSE, Mini-Mental State Examination; NA, not applicable; PD, Parkinson’s disease; UPDRS part III, Unified Parkinson’s Disease Rating Scale motor section. ^a^Pearson’s chi-squared test was used. ^b^Student’s *t*-test was used. ^c^Mann-Whitney U test was used*.

**Figure 1:**
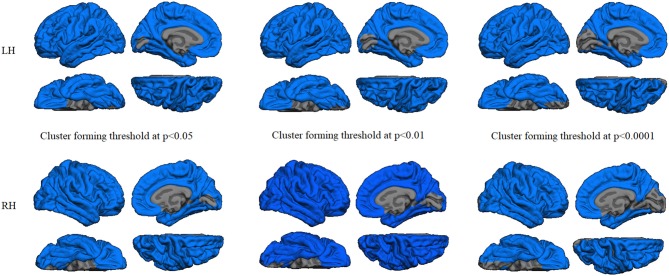
Gray/white matter contrast (GWC) correlations with age in the patients group. Color maps indicate negative correlation between age and GWC. Three levels of cluster-forming thresholds are shown. All results were corrected by Monte Carlo simulation at a cluster-wise *p* < 0.05.

**Figure 2:**
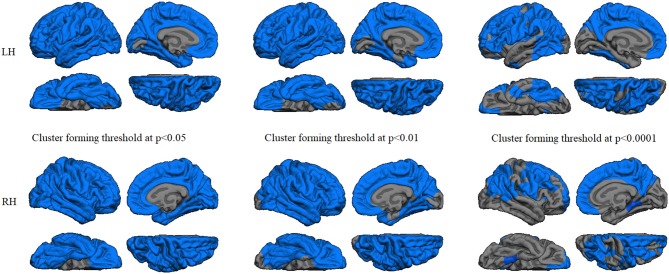
GWC correlations with age in the controls group. Color maps indicate negative correlation between age and GWC. Three levels of cluster-forming thresholds are shown. All results were corrected by Monte Carlo simulation at a cluster-wise *p* < 0.05.

Regression analyses between GWC maps and age showed significant correlations involving almost the entire brain in both HC and PD groups when applying cluster forming thresholds at *p* < 0.05 and *p* < 0.01. At the *p* < 0.0001 threshold for the HC group, the significant correlation cluster involved bilateral parahippocampal, left lateral temporal, and widespread bilateral frontal and parietal regions, whereas in the PD group the correlation also involved the entire cortex (Figures 1, 2 and Table 2). There were no significant correlations between GWC and any clinical disease-related variables.

**Table 2:** Gray/white matter contrast information from the correlations with age in PD patients and HCs.

Cortical area	Cluster size (mm^2^)	Stats	*P*-value	MNI coordinates (*x y z*)^1^
*PD patients—age correlation p < 0.05*
Left precentral	75,459.3	−16.434	0.0002	−51 −3 7
Right postcentral	74,772.5	−17.129	0.0002	47 −11 18
*PD patients—age correlation p < 0.01*
Left precentral	74,640.3	−16.434	0.0002	−51 −3 7
Right postcentral	73,577.6	−17.129	0.0002	47 −11 18
*PD patients—age correlation p < 0.0001*
Left precentral	72,108.4	−16.434	0.0002	−51 −3 7
Right postcentral	70,658.4	−17.129	0.0002	47 −11 18
*Healthy controls—age correlation p < 0.05*
Left precentral	75,239.6	−8.133	0.0002	−43 1 13
Right isthmus cingulate	75,795.5	−7.687	0.0002	4 −48 25
*Healthy controls—age correlation p < 0.01*
Left precentral	73,192.4	−8.133	0.0002	−43 1 13
Right isthmus cingulate	72,242.8	−7.687	0.0002	4 −48 25
*Healthy controls—age correlation p < 0.0001*
Left precentral	54,752.8	−8.133	0.0002	−43 1 13
Right isthmus cingulate	36,528.7	−7.687	0.0002	4 −48 25
Right parahippocampal	869.0	−6.865	0.0004	20 −37 −12

*^1^MNI305 space. Abbreviations: PD, Parkinson’s disease; MNI, Montreal Neurological Institute. Results were obtained using Monte Carlo simulation with 10,000 iterations applied to gray/white matter contrast maps to provide cluster-wise correction for multiple comparisons (1.3, 2.0 and 4.0). Significant clusters are reported at *p* < 0.05*.

**Figure 3:**
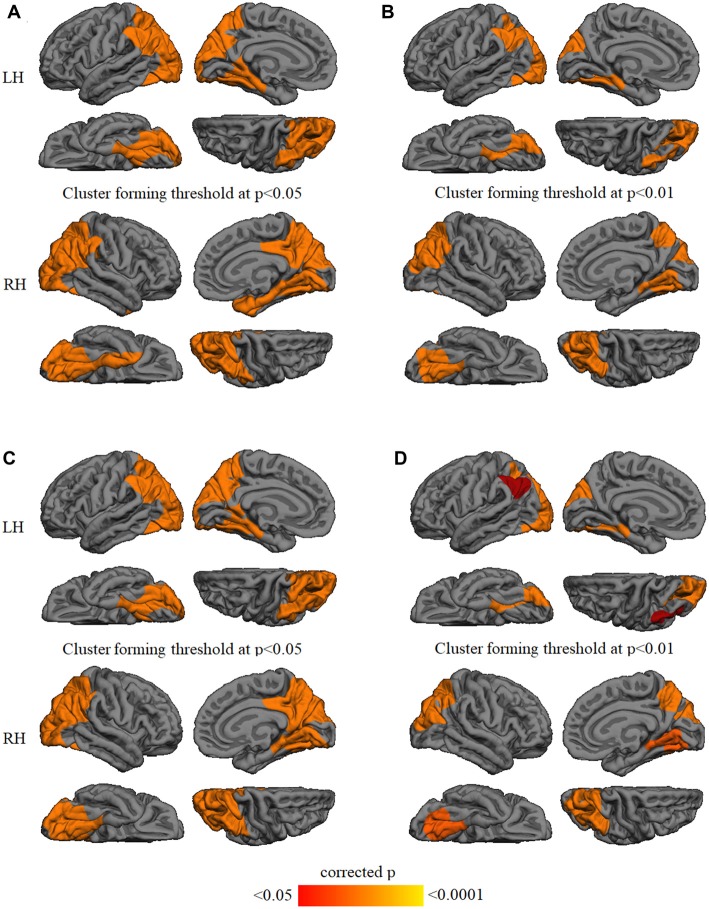
Vertex-wise comparison of cortical thickness differences with and without GWC as regressor. Color maps indicate cortical thinning significant differencesbetween Parkinson’s disease (PD) patients and controls at cluster-forming thresholds of **(A)** *p* < 0.05 and **(B)** *p* < 0.01. Color maps indicate cortical thinningsignificant differences between groups with GWC as nuisance factor at **(C)** *p* < 0.05 and **(D)** *p* < 0.01. All results were corrected by Monte Carlo simulation atcluster-wise *p* < 0.05.

GWC comparison between groups did not show significant results after Monte Carlo simulation at cluster-wise probability significance level set at any *p*-value threshold. Cortical thickness comparison between groups at the *p* < 0.05 threshold showed significant cortical thinning in PD patients, mainly in medial parieto-temporal regions including bilateral fusiform and parahippocampal gyri, cuneus, isthmus cingulate, and precuneus. Differences in dorsal cortices involved bilateral lateral occipital, as well as inferior and superior parietal cortices (Figure 3A and Table 3). At the *p* < 0.01 threshold, previous differences observed in right medial anterior temporal regions, left precuneus, and right lateral occipital were not statistically significant (Figure 3B, Table 3). Similar results were obtained after controlling this analysis for GWC (Figures 3C,D). There were no significant differences between groups at the *p* < 0.0001 cluster-forming threshold, except for a small cluster of thinning in the right lingual gyrus when using GWC as a regressor (Table 3).

**Table 3:** Cortical thickness information from the analysis between HCs and PD patients.

Cortical area	Cluster size (mm^2^)	Stats	*P*-value	MNI coordinates (*x y z*)^1^
*PD patients < healthy controls at p < 0.05*
Left superior parietal	20,272.3	3.826	0.0002	−24 −82 23
Right lingual	25,478.3	4.274	0.0002	10 −59 −0
*PD patients < healthy controls at p < 0.01*
Left superior parietal	11,282.9	3.826	0.0002	−24 −82 23
Right lingual	4059.7	4.274	0.0002	10 −59 −0
Right superior parietal	9925.9	4.256	0.0002	27 −59 47
*PD patients < healthy controls with GWC as per-vertex regressor at p < 0.05*
Left superior parietal	20,330.9	3.719	0.0002	−22 −84 21
Right lingual	22,992.3	4.531	0.0002	11 −57 −0
*PD patients < healthy controls with GWC as per-vertex regressor at p < 0.01*
Left superior parietal	8371.8	3.719	0.0002	−22 −84 21
Left supramarginal	1794.7	3.213	0.0272	−50 −52 39
Right lingual	3349.2	4.531	0.0004	11 −57 −0
Right superior parietal	101,165	4.067	0.0002	27 −60 47
*PD patients < healthy controls with GWC as per-vertex regressor at p < 0.0001*
Right lingual	208.3	4.531	0.0286	11 −57 −0

*^1^MNI305 space. Abbreviations: GWC, gray to white matter contrast; PD, Parkinson’s disease; MNI, Montreal Neurological Institute. Results were obtained using Monte Carlo simulation with 10.000 iterations applied to cortical thickness maps to provide cluster-wise correction for multiple comparisons (1.3, 2.0 and 4.0). Significant clusters were reported at *p* < 0.05*.

## Discussion

Tissue-contrast intensity showed sensitivity to aging effects. However, this measure: (1) did not differ between PD patients and the age-matched control group; (2) did not correlate with any clinical disease-related variable; and (3) the between-group differences in cortical thickness remained similar after correction for GWC.

In agreement with previous studies, we found that GWC is a very sensitive measure for the effects of aging on the brain. We showed significant correlations between GWC and age involving almost the entire cortex in both HC and PD groups. Decrease in GWC contrast related to normal aging was reported in a large sample of subjects by Salat et al. (2009). These authors also found that aging altered a large portion of the cortical mantle. Similar results were obtained in the work by Westlye et al. (2009) in a sample of 1189 normal subjects. However, these studies reported regional patterns of atrophy suggesting an increased susceptibility of frontal regions. The differences with our data could be explained by the sample characteristics; in these two previous studies, the samples included young middle-age and older adults, while all subjects in our sample were older. Frontal involvement is likely to be seen in early stages of aging. The age-related WM/GM intensity contrast decrease has been associated with the decline of WM intensity on T1-weighted images (Salat et al., 2009). A recent longitudinal study also supports this hypothesis and emphasizes the vulnerability of certain areas based on the degree of myelination (Vidal-Piñeiro et al., 2016). Assuming the hypothesis that GWC loss is due to demyelination, it might be concluded that PD is a degenerative illness without demyelination effects different from those associated with aging *per se*.

In neurodegenerative diseases such as AD, GWC decay has been found to be larger than in HC. Individuals with AD exhibited reduced GM to WM tissue contrast in several regions throughout the cortical mantle, with particularly strong effects in temporal and limbic areas (Salat et al., 2011). GWC in these areas correlated with hippocampal volumes in AD, whereas in controls the hippocampal volume was associated with GWC globally across the cortical mantle (Salat et al., 2011). Moreover, it has been reported that GWC is able to detect cerebral changes in periods of time as short as 2 years. Compared to HC, AD patients showed regional differences in the medial temporal lobes (Grydeland et al., 2013). The lack of sensitivity in PD compared with AD could be explained by the differential neuropathological basis underlying both degenerative processes. Our sample is composed of non-demented subjects; we therefore cannot rule out that GWC might be sensitive in patients with PD-related dementia. Even more interestingly, GWC might be able to distinguish demented PD patients who have comorbid AD type pathology as has been described using β-amyloid PET (Akhtar et al., 2016). In this line, Salat et al. (2011) reported that GWC changes are regionally selective to areas known to show AD pathology. GWC assessment might be insensitive to the cortical changes due to α-synuclein pathology. Another reason for the lack of sensitivity of GWC to brain atrophy in PD would be the variability of cortical thinning in PD. Other studies using cortical thickness to compare medicated PD patients and healthy subjects reported similar regions of cortical thinning than those found in the present study (Pereira et al., 2012; Garcia-Diaz et al., 2014; Segura et al., 2014; Mak et al., 2015). Posterior cortical atrophy has also been reported in de novo PD patients (Pereira et al., 2014; Uribe et al., 2018). However, in a previous study with a great part of the current sample addressed to identify patterns of cortical thinning in PD, we found that there is a subgroup of patients without manifest cortical atrophy (Uribe et al., 2016).

Moreover, previous studies showed that the inclusion of GWC as a covariate in cortical thickness analyses increased both age and diagnostic sensitivity in several regions (Westlye et al., 2009). Recently, using Alzheimer Disease Neuroimaging Initiative (ADNI) data, it has been reported that GWC did not differentiate normal subjects from MCI or AD subjects (Bauer et al., 2014). The authors only found that, after adjusting for GWC, effect sizes for the differences in cortical thickness between groups were increased.

Our results evidenced cortical thinning in PD patients in comparison with HC, mainly in temporo-parietal regions. However, we did not find significant differences in GWC, or any increased sensitivity of cortical thickness results after correction for this MRI parameter. This suggests that cortical thickness and GWC are independent measures, since differences in cortical thickness in PD vs. controls did not change after controlling for this measure. Salat et al. (2009) found that the effect of GWC changes is greater than those of morphometric changes such as cortical thinning, but also pointed out that both measures were statistically independent.

Previous studies seem to demonstrate that changes in GWC are mainly due to WM degeneration in lightly myelinated regions with age-related vulnerability. In PD, despite previous descriptions of WM abnormalities seen through DTI parameters (Hall et al., 2016; Agosta et al., 2017), changes may not be sufficient to be detected by GWC analyses when compared with healthy aging subjects. It is important to remark that the current study is cross-sectional, and it is possible that the GWC would be sensitive to degeneration over time in PD, similarly to the findings reported in AD by Grydeland et al. (2013), and also by Vidal-Piñeiro et al. (2016) in a study of normal aging that showed that longitudinal GWC changes can be detected in a healthy population over a relatively short period of time.

One strength of the present study was the utilization of different stringent cluster-forming thresholds in an attempt to identify specific regions across the cortical mantle that most strongly correlated with age. In contrast to initial studies that used T1, spin density, and T2 values of white and GM (Magnaldi et al., 1993) or segmentation algorithms (Davatzikos and Resnick, 2002) to estimate GWC, we used a cortical reconstruction approach improving the delineation of the GM/WM boundary (Dale et al., 1999). On the other hand, the lack of differences between PD patients and controls in the GWC could be due to the small sample size of controls. However, similarly to other studies using cortical thickness measures with unbalanced samples, we found significant differences between patients and controls using such measure (Lyoo et al., 2010; Pagonabarraga et al., 2013; Segura et al., 2014; Uribe et al., 2016).

In conclusion, GWC is a very sensitive measure for detecting aging effects, but may not be helpful in differentiating atrophy patterns between PD patients and controls.

## Author Contributions

CJ contributed in the design of the study. CU, AA, AIG-D and AC contributed to the analysis of the data and CU, BS, HCB, AA, AIG-D, AC, MJM, FV, YC, NB and CJ contributed to the interpretation of the data. CU, BS contributed to the draft of the article. CU, BS, HCB, AA, AIG-D, AC, MJM, FV, YC, NB, CJ revised the manuscript critically for important intellectual content and approved the final version of the manuscript.

## Conflict of Interest Statement

The authors declare that the research was conducted in the absence of any commercial or financial relationships that could be construed as a potential conflict of interest.

## Footnotes

^1^https://surfer.nmr.mgh.harvard.edu/fswiki/pctsurfcon

## References

[B1] Agosta F. Galantucci S. Filippi M. (2017). Advanced magnetic resonance imaging of neurodegenerative diseases. Neurol. Sci. 38, 41–51. 10.1007/s10072-016-2764-x 27848119

[B2] Akhtar R. S. Xie S. X. Brennan L. Pontecorvo M. J. Hurtig H. I. Trojanowski J. Q.. (2016). Amyloid-β positron emission tomography imaging of Alzheimer’s pathology in Parkinson’s disease dementia. Mov. Disord. Clin. Pract. 3, 367–375. 10.1002/mdc3.12290 27500181 PMC4971540

[B3] Bansal R. Hao X. Liu F. Xu D. Liu J. Peterson B. S. (2013). The effects of changing water content, relaxation times, and tissue contrast on tissue segmentation and measures of cortical anatomy in MR images. Magn. Reson. Imaging 31, 1709–1730. 10.1016/j.mri.2013.07.017 24055410 PMC4241465

[B4] Bauer C. Cabral H. Killiany R. Alzheimer’s Disease Neuroimaging Initiative. (2014). It is unclear if adjusting cortical thickness for changes in gray/white matter intensity ratio improves discrimination between normal aging, MCI, and AD. Brain Imaging Behav. 8, 133–140. 10.1007/s11682-013-9268-6 24535034 PMC3930075

[B6] Dale A. M. Fischl B. Sereno M. I. (1999). Cortical surface-based analysis: I. Segmentation and surface reconstruction. Neuroimage 9, 179–194. 10.1006/nimg.1998.0395 9931268

[B5] Dale A. M. Sereno M. I. (1993). Improved localizadon of cortical activity by combining EEG and MEG with MRI cortical surface reconstruction: a linear approach. J. Cogn. Neurosci. 5, 162–176. 10.1162/jocn.1993.5.2.162 23972151

[B7] Davatzikos C. Resnick S. M. (2002). Degenerative age changes in white matter connectivity visualized *in vivo* using magnetic resonance imaging. Cereb. Cortex 12, 767–771. 10.1093/cercor/12.7.767 12050088

[B8] Elton R. F. S. (1987). “UPDRS program member unified Parkinson’s disease rating scale,” in Recent Developments in Parkinson’s Disease, Vol. 2, eds Fahn D. Marsden S. Goldstein C. D. Calne M. (Florham Park, NJ: Macmillan Healthcare Information), 153–163.

[B9] Emre M. Aarsland D. Brown R. Burn D. J. Duyckaerts C. Mizuno Y.. (2007). Clinical diagnostic criteria for dementia associated with Parkinson’s disease. Mov. Disord. 22, 1689–1707; quiz 1837. 10.1002/mds.21507 17542011

[B10] Fischl B. Dale A. M. (2000). Measuring the thickness of the human cerebral cortex from magnetic resonance images. Proc. Natl. Acad. Sci. U S A 97, 11050–11055. 10.1073/pnas.200033797 10984517 PMC27146

[B11] Fischl B. Liu A. Dale A. M. (2001). Automated manifold surgery: constructing geometrically accurate and topologically correct models of the human cerebral cortex. IEEE Trans. Med. Imaging 20, 70–80. 10.1109/42.906426 11293693

[B12] Fischl B. Salat D. H. Busa E. Albert M. Dieterich M. Haselgrove C.. (2002). Whole brain segmentation: automated labeling of neuroanatomical structures in the human brain. Neuron 33, 341–355. 10.1016/S0896-6273(02)00569-X 11832223

[B13] Folstein M. F. Folstein S. E. McHugh P. R. (1975). “Mini-mental state”. A practical method for grading the cognitive state of patients for the clinician. J. Psychiatr. Res. 12, 189–198. 10.1016/0022-3956(75)90026-6 1202204

[B14] Garcia-Diaz A. I. Segura B. Baggio H. C. Marti M. J. Valldeoriola F. Compta Y.. (2014). Structural MRI correlates of the MMSE and pentagon copying test in Parkinson’s disease. Parkinsonism Relat. Disord. 20, 1405–1410. 10.1016/j.parkreldis.2014.10.014 25457818

[B15] Grydeland H. Westlye L. T. Walhovd K. B. Fjell A. M. (2013). Improved prediction of Alzheimer’s disease with longitudinal white matter/gray matter contrast changes. Hum. Brain Mapp. 34, 2775–2785. 10.1002/hbm.22103 22674625 PMC6870108

[B16] Hall J. M. Ehgoetz Martens K. A. Walton C. C. O’Callaghan C. Keller P. E. Lewis S. J. G.. (2016). Diffusion alterations associated with Parkinson’s disease symptomatology: a review of the literature. Parkinsonism Relat. Disord. 33, 12–26. 10.1016/j.parkreldis.2016.09.026 27765426

[B17] Hoehn M. M. Yahr M. D. (1967). Parkinsonism: onset, progression, and mortality. Neurology 17, 427–442. 10.1212/wnl.17.5.427 6067254

[B18] Hughes A. J. Daniel S. E. Kilford L. Lees A. J. (1992). Accuracy of clinical diagnosis of idiopathic Parkinson’s disease: a clinico-pathological study of 100 cases. J. Neurol. Neurosurg. Psychiatry 55, 181–184. 10.1136/jnnp.55.3.181 1564476 PMC1014720

[B19] Jefferson A. L. Gifford K. A. Damon S. Chapman G. W. IV. Liu D. Sparling J.. (2015). Gray and white matter tissue contrast differentiates Mild Cognitive Impairment converters from non-converters. Brain Imaging Behav. 9, 141–148. 10.1007/s11682-014-9291-2 24493370 PMC4146750

[B20] Koenig S. H. (1991). Cholesterol of myelin is the determinant of gray-white contrast in MRI of brain. Magn. Reson. Med. 20, 285–291. 10.1002/mrm.1910200210 1775053

[B21] Litvan I. Goldman J. G. Tröster A. I. Schmand B. A. Weintraub D. Petersen R. C.. (2012). Diagnostic criteria for mild cognitive impairment in Parkinson’s disease: movement disorder society task force guidelines. Mov. Disord. 27, 349–356. 10.1002/mds.24893 22275317 PMC3641655

[B22] Lyoo C. Ryu Y. Lee M. (2010). Topographical distribution of cerebral cortical thinning in patients with mild Parkinson’s disease without dementia. Mov. Disord. 25, 496–499. 10.1002/mds.22975 20108369

[B23] Magnaldi S. Ukmar M. Vasciaveo A. Longo R. Pozzi-Mucelli R. S. (1993). Contrast between white and grey matter: MRI appearance with ageing. Eur. Radiol. 3, 513–519. 10.1007/bf00169600

[B24] Mak E. Su L. Williams G. B. Firbank M. J. Lawson R. A. Yarnall A. J.. (2015). Baseline and longitudinal grey matter changes in newly diagnosed Parkinson’s disease: ICICLE-PD study. Brain 138, 2974–2986. 10.1093/brain/awv211 26173861 PMC4671477

[B25] Ogg R. J. Steen R. G. (1998). Age-related changes in brain T1 are correlated with putative iron concentration. Magn. Reson. Med. 40, 749–753. 10.1002/mrm.1910400516 9797159

[B26] Pagonabarraga J. Corcuera-Solano I. Vives-Gilabert Y. Llebaria G. García-Sánchez C. Pascual-Sedano B.. (2013). Pattern of regional cortical thinning associated with cognitive deterioration in Parkinson’s disease. PLoS One 8:e54980. 10.1371/journal.pone.0054980 23359616 PMC3554657

[B27] Pereira J. B. Ibarretxe-Bilbao N. Marti M. J. Compta Y. Junqué C. Bargallo N.. (2012). Assessment of cortical degeneration in patients with Parkinson’s disease by voxel-based morphometry, cortical folding, and cortical thickness. Hum. Brain Mapp. 33, 2521–2534. 10.1002/hbm.21378 21898679 PMC6870035

[B28] Pereira J. B. Svenningsson P. Weintraub D. Brønnick K. Lebedev A. Westman E.. (2014). Initial cognitive decline is associated with cortical thinning in early Parkinson disease. Neurology 82, 2017–2025. 10.1212/WNL.0000000000000483 24808018 PMC4105257

[B29] Salat D. H. Chen J. J. van der Kouwe A. J. Greve D. N. Fischl B. Rosas H. (2011). Hippocampal degeneration is associated with temporal and limbic gray matter/white matter tissue contrast in Alzheimer’s disease. Neuroimage 54, 1795–1802. 10.1016/j.neuroimage.2010.10.034 20965261 PMC3021138

[B30] Salat D. H. Lee S. Y. van der Kouwe A. J. Greve D. N. Fischl B. Rosas H. D. (2009). Age-associated alterations in cortical gray and white matter signal intensity and gray to white matter contrast. Neuroimage 48, 21–28. 10.1016/j.neuroimage.2009.06.074 19580876 PMC2750073

[B31] Ségonne F. Pacheco J. Fischl B. (2007). Geometrically accurate topology-correction of cortical surfaces using nonseparating loops. IEEE Trans. Med. Imaging 26, 518–529. 10.1109/tmi.2006.887364 17427739

[B32] Segura B. Baggio H. C. Marti M. J. Valldeoriola F. Compta Y. Garcia-Diaz A. I.. (2014). Cortical thinning associated with mild cognitive impairment in Parkinson’s disease. Mov. Disord. 29, 1495–1503. 10.1002/mds.25982 25100674

[B33] Tomlinson C. L. Stowe R. Patel S. Rick C. Gray R. Clarke C. E. (2010). Systematic review of levodopa dose equivalency reporting in Parkinson’s disease. Mov. Disord. 25, 2649–2653. 10.1002/mds.23429 21069833

[B34] Uribe C. Segura B. Baggio H. C. Abos A. Garcia-Diaz A. I. Campabadal A.. (2018). Cortical atrophy patterns in early Parkinson’s disease patients using hierarchical cluster analysis. Parkinsonism Relat. Disord. [Epub ahead of print]. 10.1016/j.parkreldis.2018.02.006 29449187

[B35] Uribe C. Segura B. Baggio H. C. Abos A. Marti M. J. Valldeoriola F.. (2016). Patterns of cortical thinning in nondemented Parkinson’s disease patients. Mov. Disord. 31, 699–708. 10.1002/mds.26590 27094093 PMC5061099

[B36] Vidal-Piñeiro D. Walhovd K. B. Storsve A. B. Grydeland H. Rohani D. A. Fjell A. M. (2016). Accelerated longitudinal gray/white matter contrast decline in aging in lightly myelinated cortical regions. Hum. Brain Mapp. 37, 3669–3684. 10.1002/hbm.23267 27228371 PMC6867532

[B37] Westlye L. T. Walhovd K. B. Dale A. M. Espeseth T. Reinvang I. Raz N.. (2009). Increased sensitivity to effects of normal aging and Alzheimer’s disease on cortical thickness by adjustment for local variability in gray/white contrast: a multi-sample MRI study. Neuroimage 47, 1545–1557. 10.1016/j.neuroimage.2009.05.084 19501655 PMC2828679

